# Alkoxy- and Silanol-Functionalized Cage-Type Oligosiloxanes as Molecular Building Blocks to Construct Nanoporous Materials

**DOI:** 10.3390/molecules25030524

**Published:** 2020-01-25

**Authors:** Atsushi Shimojima, Kazuyuki Kuroda

**Affiliations:** 1 Faculty of Science and Engineering, Waseda University, 3-4-1 Okubo, Shinjuku-ku, Tokyo 169-8555, Japan; 2 Kagami Memorial Research Institute for Materials Science and Technology, Waseda University, 2-8-26 Nishiwaseda, Shinjuku-ku, Tokyo 169-0051, Japan

**Keywords:** cage siloxanes, porous materials, self-assembly, hydrogen bond

## Abstract

Siloxane-based materials have a wide range of applications. Cage-type oligosiloxanes have attracted significant attention as molecular building blocks to construct novel siloxane-based nanoporous materials with promising applications such as in catalysis and adsorption. This paper reviews recent progress in the preparation of siloxane-based nanoporous materials using alkoxy- and silanol-functionalized cage siloxanes. The arrangement of cage siloxanes units is controlled by various methods, including amphiphilic self-assembly, hydrogen bonding of silanol groups, and regioselective functionalization, toward the preparation of ordered nanoporous siloxane-based materials.

## 1. Introduction

Ordered nanoporous materials have applications in various fields such as catalysis, adsorption, optics, electronics, and medicine [1,2,3]. Siloxane-based nanoporous materials are particularly important because of the abundant resources, low-toxicity, structural diversity, and high thermal stability of the siloxane (Si–O–Si) networks. Fine control of the pore structures is essential for many applications. Zeolites are microporous siloxane-based materials with a variety of crystalline frameworks [1,2,4]. However, the rational design of the zeolite frameworks is difficult, mainly due to the harsh hydrothermal conditions required for their crystallization. Mesoporous silica is another type of ordered siloxane-based nanoporous materials that is formed by self-assembly processes using surfactants and silicate species under relatively mild conditions [2,5]. The composition and mesostructure (two-dimensional (2D) hexagonal, cubic, etc.) are easily controllable; however, the pore walls of mesoporous silica are amorphous at the molecular level in almost all cases, limiting their practical applications.

**Figure 1:**
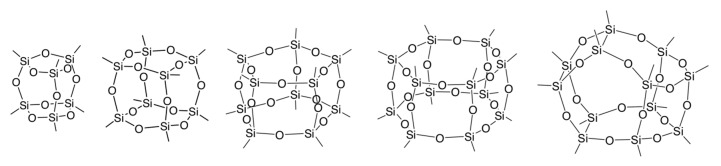
Typical structures of cage-type oligosiloxanes. The corner substituents are omitted for clarity.

The use of well-defined oligosiloxanes as molecular building blocks has received great interest in overcoming these issues [6,7]. The controlled connection of oligosiloxanes by the soft-chemical approach enables us to construct a variety of silica frameworks depending on the molecular structure and arrangement of the building blocks. Cage siloxanes are most attractive among various oligosiloxanes such as those with chain- [8,9], ring- [10,11,12], ladder- [13,14], and cage-like structures [14,15,16,17]. The rigid structures with multiple functional groups directing outward from the cage are quite useful for constructing three-dimensional (3D) porous frameworks. Cage siloxanes with various polyhedral structures are known (Figure 1) [15,16,17,18]. It is noteworthy that the double-four-ring (D4R), double-five-ring (D5R), and double-six-ring (D6R) structures are present as secondary building blocks of some zeolites. Cage-type organosiloxane oligomers containing organic groups in the frameworks are also available as building blocks [19]. For connection of cage siloxanes, various functional groups such as Si-R (R = organic groups), Si-H, Si-OR’ (R’ = alkyl), and Si-OH groups can be attached to the corner Si atoms.

Numerous efforts have been made on the synthesis of nanoporous materials by cross-linking cage siloxanes with siloxane bonds or organic linkers. This paper focuses on the construction of nanoporous siloxane frameworks by connecting cage siloxanes with siloxane bonds. In addition to the D4R siloxanes, other cage siloxanes have also been used. Among various functional groups that can be employed for the siloxane bond formation (e.g., Si-H, Si-Cl, Si-OR’, and Si-OH groups) [20,21], alkoxy (Si-OR’) groups are most commonly used for the formation of siloxane bonds by simple hydrolysis and condensation reactions. Silanol (Si-OH) groups are also important as they can readily form siloxane bonds by dehydration condensation or by silylation with chlorosilanes. Control of the arrangement and connection of silanol-modified cage siloxanes is crucial for the production of porous materials with well-defined structures.

## 2. Synthesis Routes to Cage Siloxanes as Building Blocks

**Figure 2:**
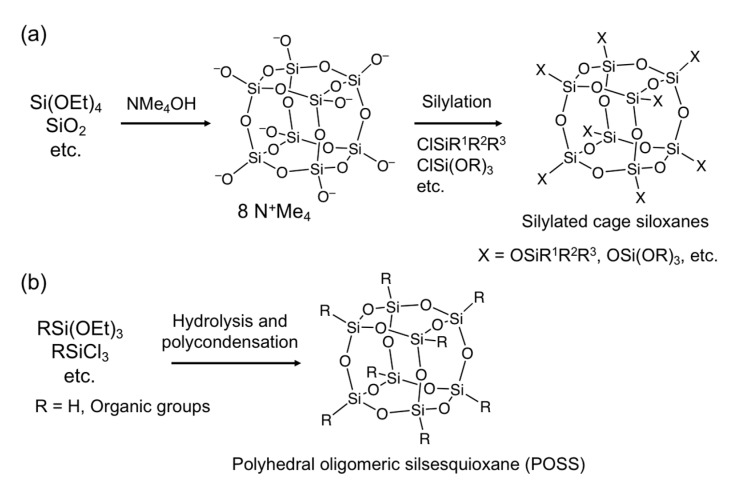
General synthetic routes to cage-type oligosiloxanes with a D4R structure. (**a**) Synthesis of D4R silicate anions using TMAOH and subsequent silylation with chlorosilane to introduce functional groups; (**b**) Synthesis of POSSs.

Cage siloxanes (XSiO_1.5_)*_n_* (X = O^–^, H, organic group; *n* = 6, 8, 10, etc.) can be obtained when the hydrolysis and polycondensation reactions of alkoxysilanes or chlorosilanes are performed under specific conditions. Anionic cage silicate with Si-O^–^ groups are formed in basic aqueous silicate solutions containing quaternary ammonium cations. For example, in the presence of tetramethylammonium (TMA) cations, cubic silicate (Si_8_O_20_^8−^) with a D4R structure is almost quantitatively formed (Figure 2a), while double-three-ring (D3R) and D5R silicates become dominant in the presence of tetraethylammonium (TEA) cations and tetrabutylammonium (TBA) cations, respectively [16,22]. In addition, D6R silicate anion (Si_12_O_30_^12−^) can be obtained as an inclusion complex with α-cyclodextrin [23,24]. Furthermore, cage-type organosiloxane oligomers can be selectively formed by hydrolysis and polycondensation of a methylene-bridged bis-trialkoxysilane [(EtO)_3_Si–CH_2_–Si(OEt)_3_] in the presence of TMAOH [19].

These cage-type silicate and organosilicate anions can be derivatized by silylation of the corner Si-O^–^ groups with chlorosilanes (Figure 2a). Oligosiloxanes having alkoxysilyl groups suitable for sol–gel reactions can be obtained by using alkoxy(chloro)silane (R_3−*m*_(R′O)*_m_*SiCl, R = organic groups, R′ = alkyl, *m* = 1–3) as the silylating agent [25]. A decrease in the amount of water in the reaction system is crucial to avoid hydrolysis and self-condensation of the silylating agents when such silylating agents with more than two hydrolyzable groups (Si-Cl, Si-OR’, and Si-H) are used. Smet et al. reported an efficient method for preparing D4R siloxane modified with functional silyl groups [26]. They used [N(*n*-C_4_H_9_)_4_]H_7_[Si_8_O_20_]·5.33H_2_O crystals for silylation. The amount of H_2_O per D4R silicate is much smaller than that in the TMA-D4R silicate hydrate crystal (65H_2_O per D4R); therefore, a bifunctional silylating agent Me_2_SiCl_2_ was successfully used to form (Si_8_O_12_)(OSiMe_2_Cl)_8_. More importantly, Nozawa et al. succeeded in the isolation of silanol-modified D4R siloxane (Si_8_O_12_)(OH)_8_ by cation exchange of (Si_8_O_20_)(NMe_4_)_8_ using Meldrum’s acid in N,N-dimethylacetamide [27]. This compound does not contain water and could be silylated with chlorosilanes without side reactions.

Cage siloxanes with Si-H or Si-R (R = organic groups) groups, called polyhedral oligomeric silsesquioxanes (POSSs), can be synthesized from trifunctional alkoxysilanes or chlorosilanes (Figure 2b). Agaskar reported that a mixture of H_8_Si_8_O_12_ and H_10_Si_10_O_15_ with D4R and D5R structures, respectively, was formed by the reaction of trichlorosilane (HSiCl_3_) in an acidic solution containing iron chloride [28]. The Si-H groups can be converted to Si-R, Si-OR’, and Si-OH groups (R, R’ = organic groups) [29,30], which makes these POSSs quite useful as the precursors of a variety of building blocks. POSSs with organic groups, such as Me, Ph, etc., can also be synthesized [15,17]. High-yield synthesis of POSSs has been achieved by the reaction of organotrialkoxysilanes in the presence of TBAF [31]. Interestingly, encapsulation of F^–^ within the D4R cage was reported for phenyl POSS [32]. In addition, cage metallosiloxanes in which some of the Si atoms are replaced with hetero elements (such as Al and Ti) are reported [16]. These compounds have attracted much attention as building blocks, especially for catalysis. Although POSSs with organic substituents (Si–C bonds) are not directly used for siloxane bond formation, some of them allow the introduction of silyl groups by post-modification.

## 3. Preparation of Porous Materials by Hydrolysis and Polycondensation of Alkoxy-Functionalized Cage Siloxanes

Alkoxy groups (Si-OR’, R’ = Me, Et, etc.) are commonly used as functional groups for the formation of siloxane bonds. Si-OR’ groups are converted to Si-OH groups by hydrolysis (Equation (1)), and a siloxane bond is formed by a dehydration condensation reaction between the Si-OH groups (Equation (2)).

≡Si–OR’ + H_2_O → ≡Si–OH + R’OH (1)

≡Si–OH + HO–Si≡ → ≡Si–O–Si≡ + H_2_O (2)

**Figure 3:**
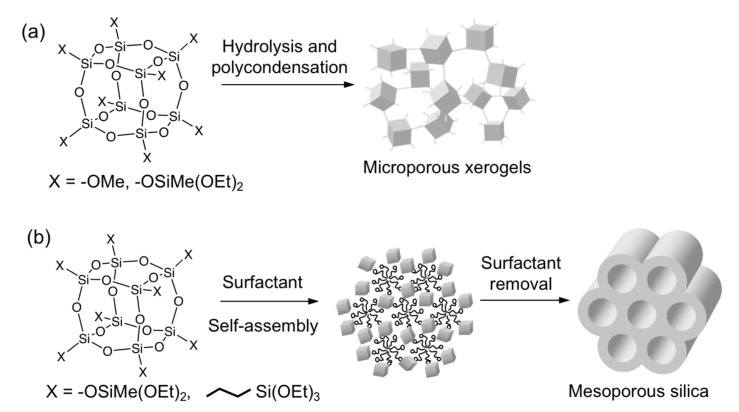
Synthesis of nanoporous materials using alkoxy-functionalized cage siloxanes: (**a**) microporous xerogels by simple sol–gel reactions and (**b**) mesoporous materials by surfactant-directed self-assembly processes.

Klemperer et al. produced a porous xerogel by hydrolysis and polycondensation reactions of a cage-type siloxane with methoxy groups (Si_8_O_12_(OMe)_8_) (Figure 3a) [33,34]. The specific surface area of the xerogel was higher than that prepared from tetramethoxysilane, suggesting that the rigid cage siloxane structure was effective for the generation of micropores. More recently, we reported the formation of a microporous xerogel by hydrolysis and polycondensation of cage siloxanes having –OSiMe(OEt)_2_ groups (Figure 3a) [25]. Solid-state ^29^Si NMR analysis suggested that the cage structure was almost retained without the cleavage of the Si–O–Si bonds. Instead of the methyl (Si-Me) groups, vinyl groups can also be incorporated into the porous network by using cage siloxane having –OSiCH=CH_2_(OiPr)_2_ groups [35]. Similar compounds with different numbers of Si-OEt groups per silyl group (Si_8_O_12_[OSiMe_2_(OEt)]_8_ and Si_8_O_12_[OSi(OEt)_3_]_8_) gave non-porous xerogels under identical conditions [25], suggesting that the degree of cross-linking is a crucial factor for the generation of voids between the cages.

The cage siloxanes with alkoxysilyl groups can be used as building blocks to produce ordered mesoporous materials by surfactant-directed self-assembly processes (Figure 3b) [25,36]. The silanol groups formed by hydrolysis of the Si-OEt groups can interact with PEO-PPO-PEO-type block copolymer surfactants to form 2D hexagonal mesostructures. When the aforementioned cage siloxane with –OSiMe(OEt)_2_ was used, the shrinkage of the mesostructure during the surfactant removal was suppressed as compared to the mesostructure prepared from a 1:1 mixture of tetraethoxysilane and methyltriethoxysilane. This is likely due to the rigid and fully condensed core of the cage siloxanes. We have also succeeded in fabricating a microporous xerogel and an ordered mesoporous material using a cage-type organosiloxane having methylene groups within the framework [37].

**Figure 4:**
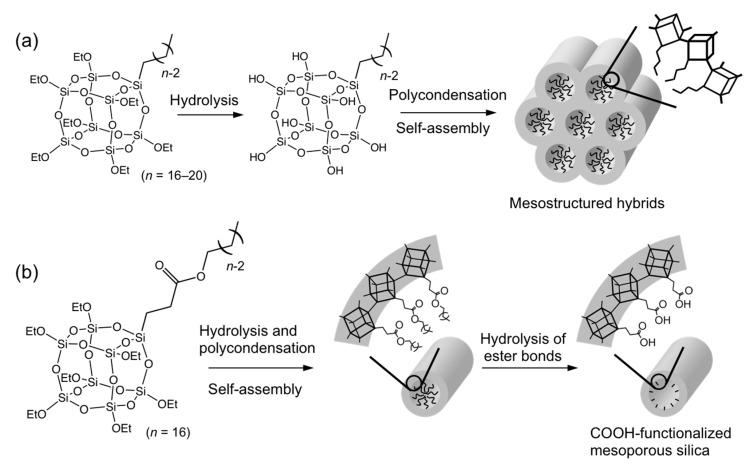
Synthesis of mesostructured materials by surfactant-free self-assembly of (**a**) cage siloxanes with a long alkyl group and (**b**) cage siloxanes with a long-chain alkyl ester group and subsequent hydrolysis of the ester bonds.

Furthermore, surfactant-free self-assembly of alkoxy-functionalized cage siloxanes can be achieved by molecular design. We reported the formation of 2D hexagonal mesostructures from alkoxy-functionalized cubic siloxanes with a long-alkyl chain (C*_n_*H_2*n*+1_Si_8_O_12_(OEt)_7_, *n* = 16, 18, and 20) (Figure 4a) [38]. The solution-state ^29^Si NMR analysis confirmed that hydrolysis of the ethoxy groups entirely proceeded without the cleavage of the Si–O–Si bonds to form amphiphilic molecules with a long-alkyl chain and seven silanol groups (C*_n_*H_2*n*+1_Si_8_O_12_(OH)_7_). Self-assembly and polycondensation of these hydrolyzed species occurred upon solvent evaporation, and alkyl chains were finally removed by calcination to form mesopores. The pore diameter was 2.8, 3.0, and 3.3 nm when the alkyl chain length was *n* = 16, 18, and 20, respectively. Similarly, mesoporous silica has been successfully produced using alkyl-substituted cage siloxanes with a D5R structure (C_16_H_33_Si_10_O_15_(OEt)_9_) [39]. This method opens a promising route to control the framework structure of mesoporous silica. However, calcination at a high temperature may cause the collapse of the cage siloxane.

One effective method to avoid thermal deterioration of the cage siloxane units by calcination is the introduction of an alkyl ester group instead of simple alkyl groups (Figure 4b) [40]. The ester bond was hydrolyzed under acidic conditions to remove alkyl chains after the formation of a mesostructure. The resulting mesoporous material contained carboxyl groups on the surface of the mesopores. The advantage of such a building block approach over the conventional method based on the post-modification is that uniform and dense distribution of organic functional groups can be achieved.

## 4. Crystalline Assemblies of Cage Siloxanes by Hydrogen Bonding of Silanol Groups

The above-mentioned microporous xerogels and mesoporous materials prepared using cage siloxanes do not have crystalline frameworks. It is necessary to establish a new methodology to achieve a regular arrangement of the cage siloxanes to construct crystalline nanoporous materials like zeolites. In the past decade, some progress has been made in the field of POSS-based inorganic–organic hybrid porous materials [41,42,43,44,45,46,47,48]. The distance and direction between the cages can be defined by introducing rigid aromatic linkers [41,42,43,44,45]. Chaikittisilp et al. reported the synthesis of a microporous material with a certain crystallinity by bridging the cage siloxanes with rigid biphenyl linkers [41]. However, it is still challenging to construct a structure with higher crystallinity by irreversible covalent bond formation reactions employed in these studies.

One promising approach to crystalline materials is solid-state polycondensation of molecular crystals. Iyoki et al. synthesized an alkoxy-functionalized cage siloxane having an adamantoxy group and seven methoxy groups [49]. Hierarchical micro- and mesoporous silica was obtained by hydrolysis and polycondensation of the methoxy groups in the solid phase, followed by the removal of the adamantoxy group by calcination. However, the initial crystalline structure has been lost during the reactions, which was probably due to the weak van der Waals interactions between the alkoxy-functionalized cage siloxanes in the molecular crystals.

Hydrogen bond (H-bond) is a relatively strong non-covalent bond widely employed for the supramolecular assembly of molecular building blocks. Modification of cage siloxanes with organic groups capable of H-bonding is effective for ordered assembly [50]. Silanol groups are more useful because of the ability to form both H-bonds and siloxane bonds. Bulky organosilanols can form molecular crystals by H-bonding of the silanol groups [51]. Kawakami et al. reported the synthesis of H-bonded, 3D crystalline networks of cage siloxanes modified with eight bulky organosilanol groups (Si_8_O_12_(CH=CHC_6_H_4_OSiPh_2_OH)_8_); however, condensation of the silanol groups was not performed [52]. Furthermore, we reported the formation of crystalline solids from cage siloxanes modified with diphenylsilanol groups (Si_8_O_12_(OSiPh_2_OH)_8_) [53]. Subsequently, solid-state condensation of the silanol groups proceeded by heating; however, structural regularities before and after the heat treatment were very low. It is likely that the bulky phenyl groups attached to the silanol groups sterically hinder the formation of highly ordered H-bonding networks and siloxane networks.

**Figure 5:**
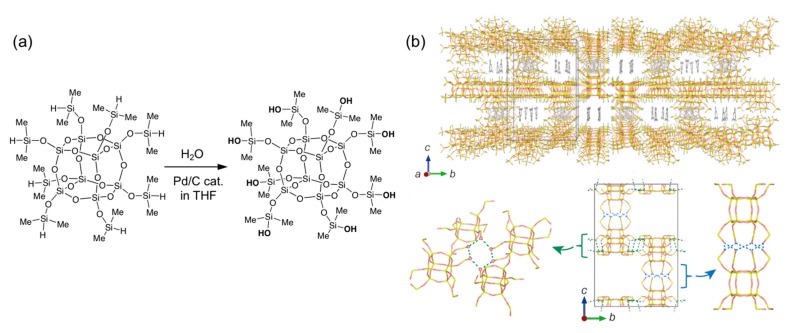
(**a**) Synthesis of dimethylsilanol-modified cage siloxane. (**b**) Structure of the molecular crystal of dimethylsilanol-modified cage siloxanes. Reproduced with permission from [55]. Copyright 2018, WILEY-VCH Verlag GmbH & Co. KGaA, Weinheim.

Recently, we found that cage siloxanes modified with dimethylsilyl (SiMe_2_H) groups were useful as precursors of the silanol modified cage siloxanes. The dimethylsilyl groups can be converted to dimethylsilanol (SiMe_2_OH) groups with water in the presence of a Pd/C catalyst without any side reactions (Figure 5a) [54,55]. It should be noted that silanol-modified cage siloxanes are hardly obtained by hydrolysis of SiMe_2_OEt groups primarily because the acid present as a hydrolysis catalyst also promotes the condensation reaction of the silanol groups.

Dimethylsilylated cubic siloxanes (Si_8_O_12_(OSiMe_2_H)_8_) can be obtained by silylation of D4R silicate anions prepared in the presence of TMA cations. After the oxidation of the Si-H groups to form Si-OH groups, the dimethylsilanol-modified cage siloxanes were crystalized from the THF/1,3,5-trimethylbenzene solution [55]. The crystals had a plate-like morphology. Single-crystal X-ray structural analysis revealed a pillared layered structure in which -SiMe_2_OH groups form both intra- and intermolecular H-bonds (Figure 5b). The solvent molecules (1,3,5-trimethylbenzene) were included in the open framework structure.

Upon heating of this molecular crystal, condensation of the silanol groups proceeded as confirmed by ^29^Si MAS NMR. The plate-like morphology was retained after the heat treatment. Although the number of the XRD peaks decreased significantly, one sharp peak was still observed, suggesting the formation of a siloxane structure that reflects the regularity of molecular crystals [54]. However, the resulting product had a low surface area, which was due to the change of the crystal structure upon the evaporation of the solvent molecules. To avoid a thermal change of the crystal structure, we performed silylation reactions to bridge the H-bonded silanol groups. A crystalline microporous material was successfully obtained by bridging the silanol groups with trichlorosilane (HSiCl_3_) [55].

A D3R siloxane modified with dimethylsilanol groups (Si_6_O_9_(OSiMe_2_OH)_6_) was also synthesized to form molecular crystals [56]. In this case, dimethylsilylation of Si_6_O_9_^6-^ at a low temperature (−94 °C) was crucial for suppressing the deterioration of the relatively unstable D3R cages. The crystal structure was different from that of the dimethylsilanol-modified D4R siloxanes. The crystal had a layered structure constructed by intermolecular H-bonding networks, and solvent (THF) molecules are included between the layers. The D3R cages were arranged into a pseudo-hexagonal structure, while the D4R cages were arranged into a tetragonal structure. These differences can be attributed to the differences in the symmetries and the distances between the adjacent silanol groups in the D3R and D4R siloxanes. However, this crystal had no open channels; therefore, a microporous material was not obtained.

H-bonded molecular crystals have also been obtained from dimethylsilanol-modified cage organosiloxane containing methylene groups in the framework [56]. Although microporous crystals have so far been obtained only from the dimethylsilanol-modified D4R siloxanes [55], regular arrangement of cage siloxanes by H-bonding of silanol groups is expected as a new soft chemical approach to the construction of crystalline zeolitic materials.

## 5. Controlled Connection of Cage Siloxanes by Regioselective Functionalization with Silanol Groups

Controlling the number and position of the reaction sites on cage siloxanes is another promising approach to construct well-defined siloxane structures. For example, mono-silanol functionalized cage siloxanes exclusively form dumbbell-like siloxanes [30,57]. Di-silanol functionalized cage siloxanes are more interesting because of their potential to form linear and cyclic polymers. Linear polymers containing the cage structure in the main chains have been synthesized by using cage siloxane possessing two reaction sites at the opposite positions [58,59].

**Figure 6:**
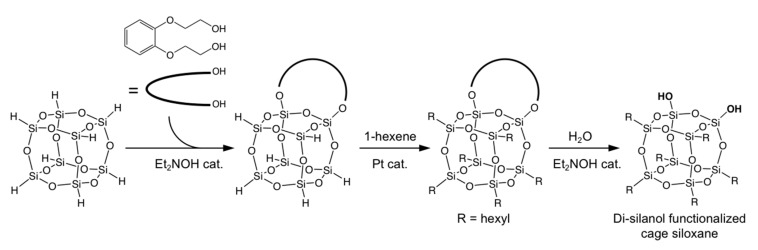
Synthesis of di-silanol functionalized cage siloxanes using a di-hydroxy organic compound.

**Figure 7:**
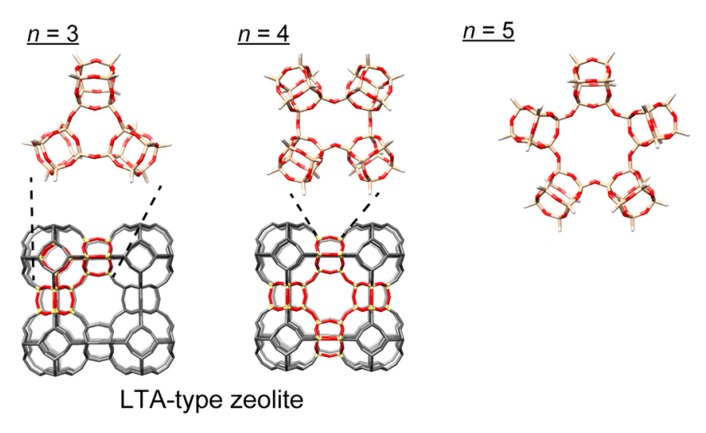
Macrocyclic compounds (cyclic-(R_6_Si_8_O_13_)*_n_*) obtained by intermolecular condensation of di-silanol functionalized D4R siloxanes. The R groups are omitted for clarity. Reprinted with permission from *Inorg. Chem.* **2018**, *57*, 14686−14691 [60]. Copyright 2018 American Chemical Society.

The control of the position of the two reaction sites is essential to form cyclic polymers of cage siloxaness. Among the three regioisomers of di-functional D4R siloxanes, the isomer having two silanol groups at adjacent corners should be the most suitable one, because of the narrow bond angle between the two silanol groups. We reported the synthesis of cage siloxanes where two adjacent corners were selectively modified with silanol groups (Figure 6) [60]. The key to this success was the use of a di-hydroxy organic compound that can bridge the adjacent corner Si atoms by the reaction with Si-H groups. After the hydrolysis of the Si-O-C bonds to form silanol groups, cyclic siloxanes with well-defined cavities (cyclic-(R_6_Si_8_O_13_)*_n_*; R = alkyl, *n* = 3, 4, and 5) were obtained by intermolecular condensation (Figure 7). These cyclic compounds can be regarded as “molecular zeolites”. The cyclic trimer and tetramer of the D4R cages can be found in the framework of LTA zeolite. These compounds are obtained as liquids and are soluble in organic solvents, providing highly accessible cavities that allow for the efficient inclusion of guest species.

## 6. Conclusions

The recent development in the construction of siloxane-based porous materials using cage-type siloxanes as molecular building blocks has been presented. Significant progress has been made on the syntheses of molecularly ordered nanoporous materials by the controlled connection of cage siloxanes based on H-bonding assembly and regioselective modification with silanol groups. This building block approach allows for the creation of novel nanoporous materials with diverse structures and compositions that cannot be achieved by conventional synthetic methods, leading to various applications.

## References

[1] Davis M.E. (2002). Ordered porous materials for emerging applications. Nature.

[2] Soler-Illia G.J.D.A.A., Sanchez C., Lebeau B., Patarin J. (2002). Chemical strategies to design textured materials: From microporous and mesoporous oxides to nanonetworks and hierarchical structures. Chem. Rev..

[3] Wang Y., Zhao Q., Han N., Bai L., Li J., Liu J., Che E., Hu L., Zhang Q., Jiang T. (2015). Mesoporous silica nanoparticles in drug delivery and biomedical applications. Nanomedicine.

[4] Li Y., Corma A., Yu J.H. (2015). Synthesis of new zeolite structures. Chem. Soc. Rev..

[5] Wan Y., Zhao D. (2007). On the controllable soft-templating approach to mesoporous silicates. Chem. Rev..

[6] Morris R.E. (2005). Modular materials from zeolite-like building blocks. J. Mater. Chem..

[7] Laine R.M. (2005). Nanobuilding blocks based on the [OSiO_1.5_]*_x_* (*x* = 6, 8, 10) octasilsesquioxanes. J. Mater. Chem..

[8] Matsumoto K., Oba Y., Nakajima Y., Shimada S., Sato K. (2018). One-pot sequence-controlled synthesis of oligosiloxanes. Angew. Chem. Int. Ed..

[9] Igarashi M., Matsumoto T., Yagihashi F., Yamashita H., Ohhara T., Hanashima T., Nakao A., Moyoshi T., Sato K., Shimada S. (2017). Non-aqueous selective synthesis of orthosilicic acid and its oligomers. Nat. Commun..

[10] Unno M., Kawaguchi Y., Kishimoto Y., Matsumoto H. (2005). Stereoisomers of 1,3,5,7-tetrahydroxy-1,3,5,7-tetraisopropylcyclotetrasiloxane: Synthesis and structures in the crystal. J. Am. Chem. Soc..

[11] Kinoshita S., Watase S., Matsukawa K., Kaneko Y. (2015). Selective synthesis of *cis*–*trans*–*cis* cyclic tetrasiloxanes and the formation of their two-dimensional layered aggregates. J. Am. Chem. Soc..

[12] Yoshikawa M., Shiba H., Wada H., Shimojima A., Kuroda K. (2018). Polymerization of cyclododecasiloxanes with Si–H and Si–OEt side groups by the Piers-Rubinsztajn reaction. Bull. Chem. Soc. Jpn..

[13] Unno M., Suto A., Matsumoto T. (2013). Laddersiloxanes—Silsesquioxanes with defined ladder structure. Russ. Chem. Rev..

[14] Unno M., Suto A., Takada K., Matsumoto H. (2000). Synthesis of ladder and cage silsesquioxanes from 1,2,3,4-tetrahydroxycyclotetrasiloxane. Bull. Chem. Soc. Jpn..

[15] Cordes D.B., Lickiss P.D., Rataboul F. (2010). Recent developments in the chemistry of cubic polyhedral oligosilsesquioxanes. Chem. Rev..

[16] Harrison P.G. (1997). Silicate cages: Precursors to new materials. J. Organomet. Chem..

[17] Lickiss P.D., Rataboul F. (2008). Fully condensed polyhedral oligosilsesquioxanes (POSS): From synthesis to application. Adv. Organomet. Chem..

[18] Tanaka K., Chujo Y. (2011). Advanced functional materials based on polyhedral oligomeric silsesquioxane (POSS). J. Mater. Chem..

[19] Shimojima A., Kuroda K. (2004). Selective formation of siloxane-based hybrid cages with methylene groups in the frameworks. Chem. Commun..

[20] Wakabayashi R., Kuroda K. (2013). Siloxane-bond formation promoted by Lewis acids: A nonhydrolytic sol–gel process and the Piers–Rubinsztajn reaction. ChemPlusChem.

[21] Kuroda K., Shimojima A., Kawahara K., Wakabayashi R., Tamura Y., Asakura Y., Kitahara M. (2014). Utilization of alkoxysilyl groups for the creation of structurally controlled siloxane-based nanomaterials. Chem. Mater..

[22] Hoebbel D., Garzó G., Engelhardt G., Vargha A. (1982). Über die konstitution und verteilung der silicatanionen in wäßrigen tetramethylammonium-silicatlösungen. Z. Anorg. Allg. Chem..

[23] Benner K., Klufers P., Schuhmacher J. (1997). A molecular composite constructed in aqueous alkaline solution from a double six-ring silicate and alpha-cyclodextrin. Angew. Chem. Int. Ed..

[24] Haouas M., Falaise C., Martineau-Corcos C., Cadot E. (2018). Cyclodextrin-driven formation of double six-ring (D6R) silicate cage: NMR spectroscopic characterization from solution to crystals. Crystals.

[25] Hagiwara Y., Shimojima A., Kuroda K. (2008). Alkoxysilylated-derivatives of double-four-ring silicate as novel building blocks of silica-based materials. Chem. Mater..

[26] Smet S., Verlooy P., Duerinckx K., Breynaert E., Taulelle F., Martens J.A. (2017). Double-four-ring [Si_8_O_12_][OH]_8_ cyclosilicate and functionalized spherosilicate synthesis from [N(*n*-C_4_H_9_)_4_]H_7_[Si_8_O_20_]·5.33H_2_O cyclosilicate hydrate crystals. Chem. Mater..

[27] Nozawa T., Matsumoto T., Yagihashi F., Beppu T., Sato K., Igarashi M. (2018). [Si_8_O_12_][OH]_8_: Isolation, structure, and reactivity of a cubic octamer of orthosilicic acid. Chem. Lett..

[28] Agaskar P.A. (1991). New synthetic route to the hydridospherosiloxanes *O*_h_-H_8_Si_8_O_12_ and *D*_5h_-H_10_Si_10_O_15_. Inorg. Chem..

[29] Bassindale A.R., Gentle T. (1996). Derivatisation of octasilsesquioxane with alcohols and silanols. J. Organomet. Chem..

[30] Saito S., Yamasue N., Wada H., Shimojima A., Kuroda K. (2016). Cubic siloxanes with both Si–H and Si–O*t*Bu groups for site-selective siloxane bond formation. Chem. Eur. J..

[31] Bassindale A.R., Liu Z., MacKinnon I.A., Taylor P.G., Yang Y., Light M.E., Horton P.N., Hursthouse M.B. (2003). A higher yielding route for T_8_ silsesquioxane cages and X-ray crystal structures of some novel spherosilicates. Dalton Trans..

[32] Bassindale A.R., Pourny M., Taylor P.G., Hursthouse M.B., Light M.E. (2003). Fluoride-ion encapsulation within a silsesquioxane cage. Angew. Chem. Int. Ed..

[33] Day V.W., Klemperer W.G., Mainz V.V., Millar D.M. (1985). Molecular building blocks for the synthesis of ceramic materials: [Si_8_O_12_](OCH_3_)_8_. J. Am. Chem. Soc..

[34] Cagle P.C., Klemperer W.G., Simmons C.A. (1990). Molecular architecture and its role in silica sol–gel polymerization. Mater. Res. Soc. Symp. Proc..

[35] Hagiwara Y., Shimojima A., Kuroda K. (2010). Formation of reactive microporous networks from alkoxyvinylsilylated siloxane cages. Bull. Chem. Soc. Jpn..

[36] Zhang L., Abbenhuis H.C.L., Yang Q., Wang Y.-M., Magusin P.C.M.M., Mezari B., Santen R.A., Li C. (2007). Mesoporous organic–inorganic hybrid materials built using polyhedral oligomeric silsesquioxane blocks. Angew. Chem. Int. Ed..

[37] Kuge H., Hagiwara Y., Shimojima A., Kuroda K. (2008). Oligomeric alkoxysilanes with cagelike hybrids as cores: Designed precursors of nanohybrid materials. Chem. Asian J..

[38] Shimojima A., Goto R., Atsumi N., Kuroda K. (2008). Self-assembly of alkyl-substituted cubic siloxane cages into ordered hybrid materials. Chem. Eur. J..

[39] Shimojima A., Kuge H., Kuroda K. (2011). Synthesis of mesostructured silica from monoalkyl-substituted double five-ring units. J. Sol-Gel Sci. Technol..

[40] Goto R., Shimojima A., Kuge H., Kuroda K. (2008). A hybrid mesoporous material with uniform distribution of carboxy groups assembled from a cubic siloxane-based precursor. Chem. Commun..

[41] Chaikittisilp W., Sugawara A., Shimojima A., Okubo T. (2010). Microporous hybrid polymer with a certain crystallinity built from functionalized cubic siloxane cages as a singular building unit. Chem. Mater..

[42] Roll M.F., Kampf J.W., Kim Y., Yi E., Laine R.M. (2010). Nano building blocks via iodination of [PhSiO_1.5_]*_n_*, forming [*p*-I-C_6_H_4_SiO_1.5_]*_n_* (*n* = 8, 10, 12), and a new route to high-surface-area, thermally stable, microporous materials via thermal elimination of I_2_. J. Am. Chem. Soc..

[43] Kim Y., Koh K., Roll M.F., Laine R.M., Matzger A.J. (2010). Porous networks assembled from octaphenylsilsesquioxane building blocks. Macromolecules.

[44] Peng Y., Ben T., Xu J., Xue M., Jing X., Deng F., Qiu S., Zhu G. (2011). A covalently-linked microporous organic-inorganic hybrid framework containing polyhedral oligomeric silsesquioxane moieties. Dalton Trans..

[45] Wu Y., Wang D., Li L., Yang W., Feng S., Liu H. (2014). Hybrid porous polymers constructed from octavinylsilsesquioxane and benzene via Friedel–Crafts reaction: Tunable porosity, gas sorption, and postfunctionalization. J. Mater. Chem. A.

[46] Chaikittisilp W., Kubo M., Moteki T., Sugawara-Narutaki A., Shimojima A., Okubo T. (2011). Porous siloxaneorganic hybrid with ultrahigh surface area through simultaneous polymerization-destruction of functionalized cubic siloxane cages. J. Am. Chem. Soc..

[47] Du Y., Ge M., Liu H. (2019). Porous polymers derived from octavinylsilsesquioxane by cationic polymerization. Macromol. Chem. Phys..

[48] Ge M., Liu H. (2018). Fluorine-containing silsesquioxane-based hybrid porous polymers mediated by bases and their use in water remediation. Chem. Eur. J..

[49] Iyoki K., Sugawara-Narutaki A., Shimojima A., Okubo T. (2013). Hierarchical porous silicavia solid-phase hydrolysis/polycondensation of cubic siloxane-based molecular units. J. Mater. Chem. A.

[50] Voisin D., Flot D., Van der Lee A., Dautel O.J., Moreau J.J.E. (2017). Hydrogen bond-directed assembly of silsesquioxanes cubes: synthesis of carboxylic acid POSS derivatives and the solid state structure of octa[2-(*p*-carboxyphenyl)ethyl] silsesquioxane. CrysEngComm.

[51] Chandrasekhar V., Boomishankar R., Nagendran S. (2004). Recent developments in the synthesis and structure of organosilanols. Chem. Rev..

[52] Kawakami Y., Sakuma Y., Wakuda T., Nakai T., Shirasaka M., Kabe Y. (2010). Hydrogen-bonding 3D networks by polyhedral organosilanols: Selective inclusion of hydrocarbons in open frameworks. Organometallics.

[53] Kawahara K., Tachibana H., Hagiwara Y., Kuroda K. (2012). A spherosilicate oligomer with eight stable silanol groups as a building block of hybrid materials. New J. Chem..

[54] Sato N., Kuroda Y., Abe T., Wada H., Shimojima A., Kuroda K. (2015). Regular assembly of cage siloxanes by hydrogen bonding of dimethylsilanol groups. Chem. Commun..

[55] Sato N., Kuroda Y., Wada H., Shimojima A., Kuroda K. (2018). Preparation of Siloxane-based microporous crystal from hydrogen bonded molecular crystal of cage siloxane. Chem. Eur. J..

[56] Sato N., Tochigi K., Kuroda Y., Wada H., Shimojima A., Kuroda K. (2019). Synthesis and crystal structure of double-three ring (D3R)-type cage siloxanes modified with dimethylsilanol groups. Dalton Trans..

[57] Anderson E., Mitchell C., Haddad T.S., Vij A., Schwab J.J., Bowers M.T. (2006). Structural characterization of POSS siloxane dimer and trimer. Chem. Mater..

[58] Hoque A., Kakihana Y., Shinke S., Kawakami Y. (2009). Polysiloxanes with periodically distributed isomeric double-decker silsesquioxane in the main chain. Macromolecules.

[59] Katsuta N., Yoshimatsu M., Komori K., Natsuaki T., Suwa K., Sakai K., Matsuo T., Ohba T., Uemura S., Watanabe S. (2017). Necklace-shaped dimethylsiloxane polymers bearing polyhedral oligomeric silsesquioxane cages with alternating length chains. Polymer.

[60] Saito S., Wada H., Shimojima A., Kuroda K. (2018). Synthesis of zeolitic macrocycles using site-selective condensation of regioselectively difunctionalized cubic siloxanes. Inorg. Chem..

